# Nomogram Predicts the Role of Contralateral Prophylactic Mastectomy in Male Patients With Unilateral Breast Cancer Based on SEER Database: A Competing Risk Analysis

**DOI:** 10.3389/fonc.2021.587797

**Published:** 2021-04-29

**Authors:** Kunlong Li, Bin Wang, Zejian Yang, Ren Yu, Heyan Chen, Yijun Li, Jianjun He, Can Zhou

**Affiliations:** ^1^ Department of Breast Surgery, First Affiliated Hospital, Xi’an Jiaotong University, Xi’an, China; ^2^ School of Medicine, Xi’an Jiaotong University, Xi’an, China

**Keywords:** male breast cancer, contralateral prophylactic mastectomy, SEER, competing risk analysis, nomogram

## Abstract

**Background:**

Contralateral prophylactic mastectomy (CPM) in female breast cancer (FBC) is supported by multiple clinical studies and consensus guidelines, but knowledge of preventive contralateral mastectomy in male breast cancer (MaBC) is very limited and its benefits are still controversial.

**Methods:**

A retrospective cohort study was enrolled with 4,405 MaBC patients who underwent unilateral mastectomy (UM) or CPM from the Surveillance, Epidemiology, and End Results (SEER) database from 1998 to 2015. A nomogram was built based on the corresponding parameters by competing risks regression to predict the 3-year, 5-year, and 8-year probabilities of BCSD (breast cancer-specific death). C-index and calibration curves were chosen for validation. Net reclassification index (NRI) and integrated discrimination improvement (IDI) were used to estimate the nomogram’s clinical utility.

**Results:**

A total of 4,197 patients received UM and 208 patients received CPM, with 63-months median follow-up. In the competing risks regression, six variables (surgery, marital status, T-stage, N-stage, histology, tumor grade) were significantly associated with BCSD. Based on these independent prognosis factors, a nomogram model was constructed. The C-index 0.75 (95%CI: 0.73-0.77) in the training cohort and 0.73 (95%CI: 0.71-0.74) in the internal validation group suggested robustness of the model. In addition, the calibration curves exhibited favorably. The NRI values (training cohort: 0.54 for 3-year, 0.55 for 5-year, and 0.49 for 8-year BCSD prediction; validation cohort: 0.51 for 3-year, 0.45 for 5-year, and 0.33 for 8-year BCSD prediction) and IDI values (training cohort: 0.02 for 3-year, 0.03 for 5-year, and 0.04 for 8-year BCSD prediction; validation cohort: 0.02 for 3-year, 0.04 for 5-year, and 0.04 for 8-year BCSD prediction) indicated that the model performed better than the AJCC criteria-based tumor staging alone.

**Conclusions:**

The administration of CPM was associated with the decrease in risk of BCSD in patients with MaBC. The nomogram could provide a precise and personalized prediction of the cumulative risk in patients with MaBC after CPM.

## Introduction

Contralateral prophylactic mastectomy (CPM) is a controversial but hot topic in the world. The application of CPM could reduce risk of contralateral breast cancer (CBC) for female patients with unilateral breast cancer (1–5). However, almost all prospective clinical trials concerning CPM are conducted in female breast cancer (FBC) patients. Consequently, the benefit of CPM on male breast cancer (MaBC) patients remains unknown due to its rarity (6).

As a rare primary breast malignancy, MaBC accounts for less than 1% of all breast cancers (7–10). Compared with FBC, previous studies suggested that patients with MaBC had different biological characteristics such as advanced age, a higher percentage of lymph node metastases, and were estrogen receptor-positive (ER+) (9, 11, 12). In contrast to FBC, MaBC tends to present BRCA2 mutation rather than BRCA1 mutation (13). Therefore, more clinical evidence for surgical strategies and subsequent treatment methods are needed for MaBC patients since current guidelines are based on female clinic data.

To further explore and identify the curative effects of CPM in patients with resectable MaBC, we followed a large cohort of males with MaBC from 1998 to 2015 from the population-based database Surveillance, Epidemiology, and End Results (SEER) cancer registry program. In the study, we established a competing risks nomogram to predict and identify those patients who could benefit from CPM.

## Materials and Methods

### Data Resource

The recent version of the SEER 18 registries’ custom data (with additional treatment fields) was used as the data source for the present population-based investigation. This database consists of 18 population-based cancer registries and covers approximately 26% of the US population across several geographic regions (14). SEER*-Stat Software version 8.3.6 (https://seer.cancer.gov/seerstat/) (Information Management Service, Inc. Calverton, MD, USA) was used to generate the case listing. All procedures were performed in accordance with approved guidelines. This study was approved by the Ethics Committee of the First Affiliated Hospital of Xi’an Jiaotong University. Informed patient consent was not required to access and use SEER data.

### Patient Cohort

Male patients diagnosed with unilateral breast cancer from 1998 to 2015 were enrolled in the study. Patients were included by following criteria: 1) primary breast cancer; 2) TNM (Breast-Adjusted American Joint Committee on Cancer, AJCC 6th) stages 0, I, II, or III; and 3) unilateral mastectomy (UM) or CPM. The demographic and clinicopathological variables were shown as follows: sex (male), age, race, site, behavior years of diagnosis, tumor grade, tumor T stage, tumor N stage, type of surgery, radiotherapy, chemotherapy, ER status, PR status, survival months, vital status, reasons of death, marital status, and breast-adjusted AJCC 6th TNM stage.

**Figure 1:**
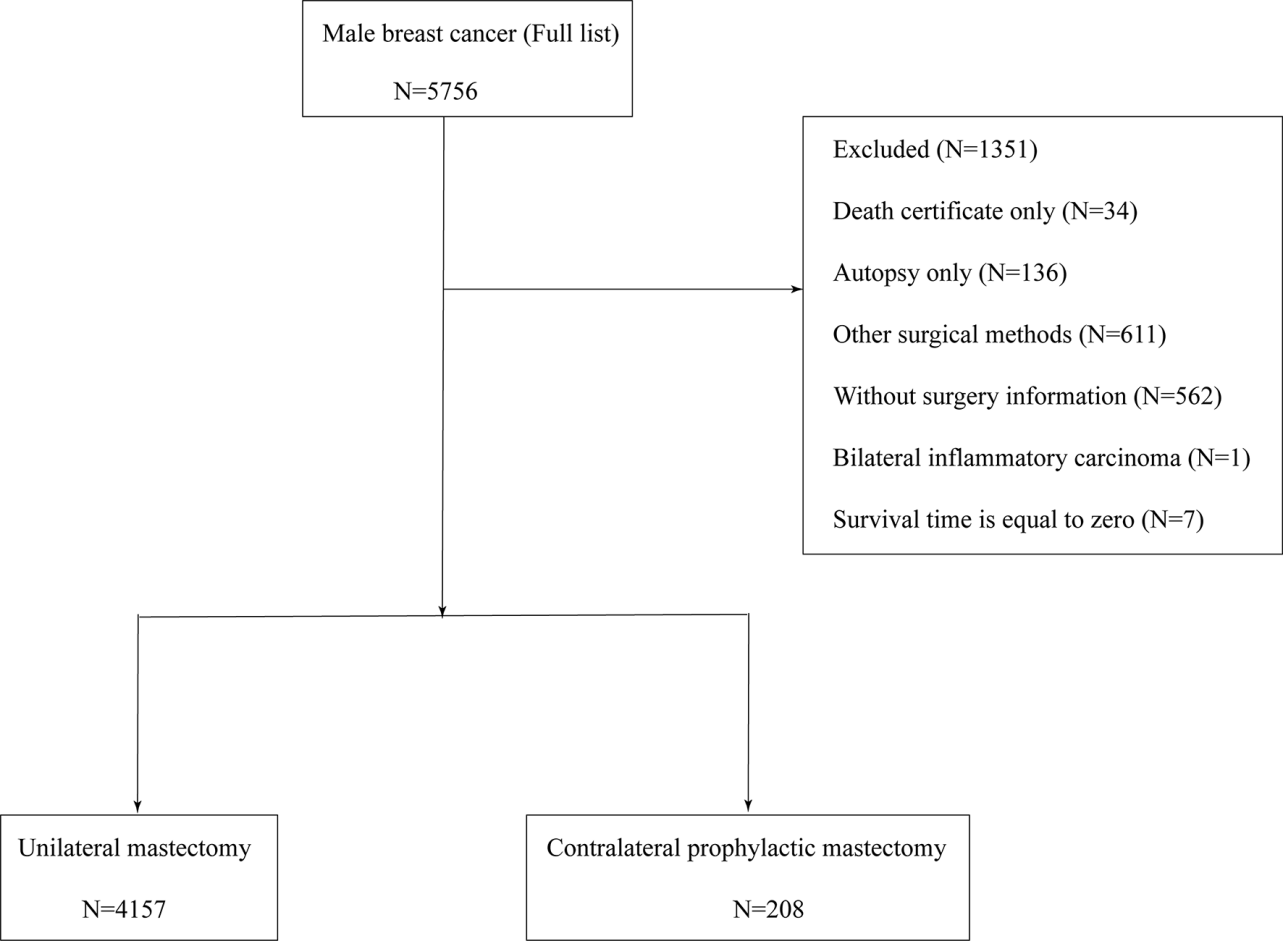
Eligibility, inclusion, and exclusion criteria of study population.

After the preliminary selection, patients were excluded by following criteria: (1) unknown AJCC stage; (2) the follow-up type of autopsy or death certificate; (3) distant metastasis (M1); (4) aged below 20 years; (5) missing surgical records; and (6) survival months is zero. **Figure 1** shows the entire screening process.

In total, 4,405 patients with MaBC were included in our cohort. To estimate the impact of CPM on prognosis, the study cohort was classified into two groups by different operation selections: UM group and CPM group. “No radiation and/or cancer-directed surgery” were regarded as no radiotherapy. “No/Unknown” chemotherapy records were regarded as no chemotherapy.

### End Points

Patients were followed up until November 2015, and the median follow-up was 63 months (ranging from 1 month to 227 months). The primary indexes, breast cancer-specific death (BCSD) and breast cancer-specific survival (BCSS), were defined as the time interval between the date of diagnosis and death due to breast cancer. The secondary outcome measurement was overall survival (OS) which was deemed as the interval from the date of diagnosis to the date of death for any reason.

### Statistical Analysis

All analyses were performed by using R statistical software version 3.6.3 (https://www.r-project.org). We used descriptive statistics to summarize demographic and clinical variables, continuous variables with normal distribution were described as means and standard deviations, categorical variables were compared using Chi-squared test or Fisher’s exact test as appropriate. Firstly, Kaplan-Meier curves and log-rank test were performed to determine the statistical differences among groups of overall survival (OS) and breast cancer-specific survival (BCSS). Secondly, a Cox proportional hazards model was constructed to find prognostic factors of MaBC by the R package of rms. Thirdly, the competing risk analysis model was used to estimate the hazard of the cumulative incidence function while controlling for the competing risks of death, which predicted BCSD by the R package of cmprsk and competing risks regression (15, 16). Fourthly, in order to predict the prognosis of MaBC after three, five, and eight years, based on the coefficients from the competing risks regression models, a nomogram was built by the R packages mstate and regplot (17). Lastly, during the validation process, concordance indexes (C-index) and calibration curves were used to determine predictive accuracy and discriminability. Net reclassification index (NRI) and integrated discrimination improvement (IDI) were performed to estimate the nomogram’s clinical utility compared with the AJCC-TNM stage system. All *P-*values were bilateral and *P*< 0.05 was considered to be statistically significant.

## Result

### Baseline Characteristics

Among the 4,405 patients from our study cohort, 95.3% (4,197/4,405) of patients received UM, while 4.7% (208/4,405) had CPM. Among these men, 82.5% of patients were white, 52.7% of patients had moderate differentiated tumors, 85% of patients had infiltrating duct carcinoma, 49.5% of patients were in the early T-stage (T0 and T1), 56.2% of patients were in the N0 stage, 23.7% of patients received chemotherapy and 38% of patients received radiation, 90.8% of patients were ER-positive (ER+), 81.2% of patients were PR-positive (PR+), and 69.7% of patients were married. Compared with patients who received UM, patients who received CPM were younger in age (59 ± 12 years versus 67 ± 12 years), and more likely to receive chemotherapy (49% versus 37.4%), while the ratio of T2 stage (38.5% versus 41.2%) and grade II (44.7% versus 53.1%) were lower. There were no statistical significance in race, histology, N stage, received radiation, ER status, PR status, and marital status. Detailed information is shown in **Table 1**.

**Table 1:** The baseline characteristics of patients with different surgery procedures in the SEER database.

Items	Total		CPM		UM		χ^2^	P-value
	N	%	N	%	N	%		
	4405	100	208	4.7	4197	95.3		
**Age (mean ± SD)**	66.98 ± 12.26		59.26 ± 12.34		67.36 ± 12.13			<0.001
**Race**							5.9	0.052
White	3633	82.5	177	85.1	3456	82.3		
Black	551	12.5	28	13.5	523	12.5		
Other/unknown	221	5.0	3	1.4	218	5.2		
**Grade**							6.58	0.04
I	540	12.3	34	16.3	506	12		
II	2322	52.7	93	44.7	2229	53.1		
III or IV	1543	35.0	81	38.9	1462	34.8		
**Histology**							1.09	0.3
Infiltrating duct carcinoma	3743	85.0	171	82.2	3572	85.1		
Other	662	15.0	37	17.8	625	14.9		
**AJCC 6th T**							10.33	0.02
T0-1	2181	49.5	112	53.8	2069	49.3		
T2	1809	41.1	80	38.5	1729	41.2		
T3	114	2.6	10	4.8	104	2.5		
T4	301	6.8	6	2.9	295	7		
**AJCC 6th N**							0.82	0.85
N0	2476	56.2	115	55.3	2361	56.3		
N1	1306	29.6	61	29.3	1245	29.7		
N2	412	9.4	23	11.1	389	9.3		
N3	211	4.8	9	4.3	202	4.8		
**Radiation**							0.4	0.84
Yes	1044	23.7	51	24.5	993	23.7		
No	3361	76.3	157	75.5	3204	76.3		
**Chemotherapy**							10.9	0.001
Yes	1672	38	102	49	1570	37.4		
No	2733	62	106	51	2627	63.6		
**ER status**							2.61	0.27
Negative	110	2.5	7	3.4	103	2.5		
Positive	3998	90.8	192	92.3	3806	90.7		
Unknown/other	297	6.7	9	4.3	288	6.9		
**PR status**							1.16	0.56
Negative	461	10.5	25	12	436	10.4		
Positive	3578	81.2	169	81.2	3409	81.2		
Unknown/other	366	8.3	14	6.7	352	8.4		
**Marital status**							0.15	0.93
Married	3070	69.7	143	68.8	2927	69.7		
Single	1158	26.3	57	27.4	1101	26.2		
Unknown	177	4	8	3.8	169	4.1		

SEER, Surveillance, Surveillance, Epidemiology, and End Results; AJCC, American Joint Committee on Cancer; ER, estrogen receptor; PR, progesterone receptor; BCSD, breast cancer-specific death; OCSD, other cause-specific death; UM, unilateral mastectomy; CPM, contralateral prophylactic mastectomy.

### Kaplan–Meier Analysis of OS and BCSS

**Figure 2:**
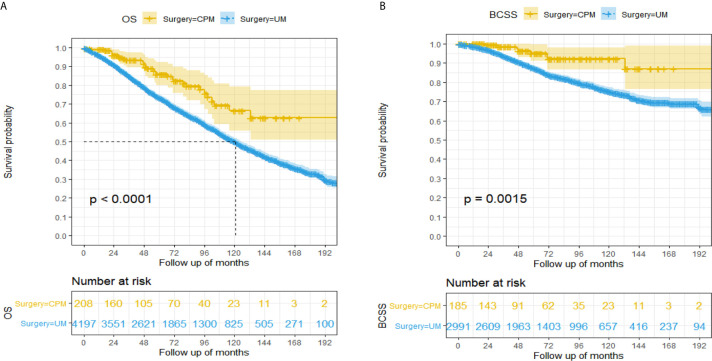
Kaplan-Meier survival analysis for male breast cancer patients. **(A)** Overall survival curves in the CPM group and UM group. **(B)** Breast cancer-specific survival curves in the CPM group and UM group.

A total of 1,757 (39.89%) patients died in this cohort study, and 30.05% (528/1,757) of them had a breast cancer‐specific death, while 69.95% (1,229/1,757) did not. The OS after three, five, and eight years was 93.3%, 85.9%, and 75.7% in the CPM group, respectively; and 84.9%, 73.3% and 59.4% in the UM group, respectively (**Figure 2A**). The BCSS after three, five, and eight years was 98.5%, 95.1%, and 92.1% in the CPM group, respectively; and 93.7%, 87.3% and 79.8% in the UM group, respectively (**Figure 2B**).

The hazard ratio (HR) summarized the risk of OS and BCSS. As shown in **Figures 2A**, **B**, the CPM group was significantly correlated with better OS (HR=0.48, 95%CI: 0.34-0.69, *P*<0.001) and BCSS (HR=0.34, 95%CI: 0.17-0.68, *P*<0.001) in comparison with the UM group.

### Univariate and Multivariate Cox Regression Model Analysis of MaBC Patients

As shown in **Table 2**, through univariate Cox analysis, a total of nine variables, such as age, race, histology, tumor grade, T-stage, N-stage, surgery, receiving chemotherapy, and marital status, were significantly associated with OS and BCSS. To further explore the independent predictive consequences of OS and BCSS, multivariate Cox proportional hazard regression analyses were performed. After adjustment of the clinical features in the Cox model, CPM was only significantly correlated with better BCSS (HR=0.44, 95%CI: 0.22-0.89, *P*=0.02) and threshold value of OS (HR, 0.72, 95% CI: 0.51-1.02, *P*=0.07). In addition, race, tumor grade, histology, tumor T stage, tumor N stage, age, and marital status were independent predictive factors in OS and BCSS.

**Table 2:** Univariate and multivariate Cox regression model analysis of MaBC patients.

Characteristics	OS						BCSS					
	Univariate analysis			Multivariate analysis			Univariate analysis			Multivariate analysis		
	Hazard ratio	95% CI	*P*-value	Hazard ratio	95% CI	*P*-value	Hazard ratio	95% CI	*P*-value	Hazard ratio	95% CI	*P*-value
**Age**	1.06	1.05-1.07	<0.001	1.06	1.05-1.07	<0.001	1.02	1.01-1.03	0.001	1.02	1.01-1.03	<0.001
**Race**												
White	as reference			as reference			as reference			as reference		
Black	1.19	1.03-1.36	0.02	1.39	1.21-1.6	<0.001	1.46	1.16-1.84	0.001	1.34	1.05-1.7	0.020
Other/unknown	0.64	0.49-0.84	0.001	0.82	0.63-1.08	0.15	0.64	0.4-1.03	0.07	0.70	0.44-1.12	0.14
**Histology**												
Other	as reference			as reference			as reference			as reference		
Infiltrating duct carcinoma	1.16	1.01-1.33	0.03	1.17	1.02-1.35	0.02	1.62	1.23-2.14	<0.001	1.41	1.06-1.87	0.02
**Grade**												
I	as reference			as reference			as reference			as reference		
II	1.23	1.05-1.44	0.01	1.11	0.94-1.3	0.22	2.21	1.48-3.29	<0.001	1.67	1.11-2.49	0.01
III or IV	1.59	1.35-1.87	<0.001	1.42	1.19-1.67	<0.001	4.14	2.79-6.15	<0.001	2.61	1.75-3.91	<0.001
**AJCC 6th T**												
0-I	as reference			as reference			as reference			as reference		
II	1.59	1.44-1.76	<0.001	1.39	1.25-1.54	<0.001	3.01	2.47-3.68	<0.001	2.08	1.69-2.56	<0.001
III	1.81	1.38-2.37	<0.001	1.65	1.25-2.17	<0.001	4.29	2.82-6.5	<0.001	2.94	1.91-4.53	<0.001
IV	2.78	2.39-3.26	<0.001	1.09	1.76-2.48	<0.001	6.26	4.71-8.31	<0.001	3.54	2.61-4,81	<0.001
**AJCC 6th N**												
0	as reference			as reference			as reference			as reference		
I	1.14	1.03-1.27	0.02	1.81	1.44-2.27	<0.001	2.17	1.75-2.68	<0.001	1.88	1.49-2.35	<0.001
II	1.65	1.42-1.92	<0.001	2.85	2.16-3.76	<0.001	4.39	3.43-5.62	<0.001	3.30	2.49-4.37	<0.001
III	2.06	1.7-2.5	<0.001	4.99	3.68-6.78	<0.001	7.28	6.57-9.53	<0.001	4.90	2.66-6.59	<0.001
**Surgery**												
UM	as reference			as reference			as reference			as reference		
CPM	0.48	0.34-0.69	<0.001	0.72	0.51-1.02	0.07	0.002	0.17-0.68	<0.001	0.44	0.22-0.89	0.02
**Radiation**												
No	as reference						as reference			as reference		
Yes	1.07	0.96-1.19	0.23				1.87	1.59-2.23	<0.001	0.86	0.7-1.06	0.17
**Chemotherapy**												
No	as reference			as reference			as reference			as reference		
Yes	0.70	0.63-0.77	<0.001	0.79	0.71-0.9	<0.001	1.53	1.29-1.82	<0.001	0.92	0.74-1.14	0.45
**ER status**												
Other	as reference						as reference					
Negative	1.17	0.86-1.58	0.32				1.62	0.97-2.72	0.07			
Positive	0.87	0.75-1.02	0.10				0.90	0.65-1.24	0.52			
**PR status**												
Unknown/other	as reference						as reference					
Negative	1.02	0.84-1.24	0.83				1.39	0.98-1.97	0.06			
Positive	0.91	0.78-1.05	0.20				0.90	0.67-1.2	0.46			
**Marital status**												
Married	as reference			as reference			as reference			as reference		
Single	1.59	1.43-1.76	<0.001	1.45	1.31-1.61	<0.001	2.03	1.69-2.42	<0.001	1.69	1.41-2.04	<0.001
Unknown	0.96	0.73-1.25	0.75	1.02	0.78-1.34	0.87	0.70	0.39-1.25	0.23	0.88	0.49-1.57	0.67

SEER, Surveillance, Surveillance, Epidemiology, and End Results; AJCC, American Joint Committee on Cancer; ER, estrogen receptor; PR, progesterone receptor; OS, overall survival; BCSS, breast cancer-specific death; UM, unilateral mastectomy; CPM, contralateral prophylactic mastectomy.

### Nomogram Variable Screening by Competing Risk Analysis

**Figure 3:**
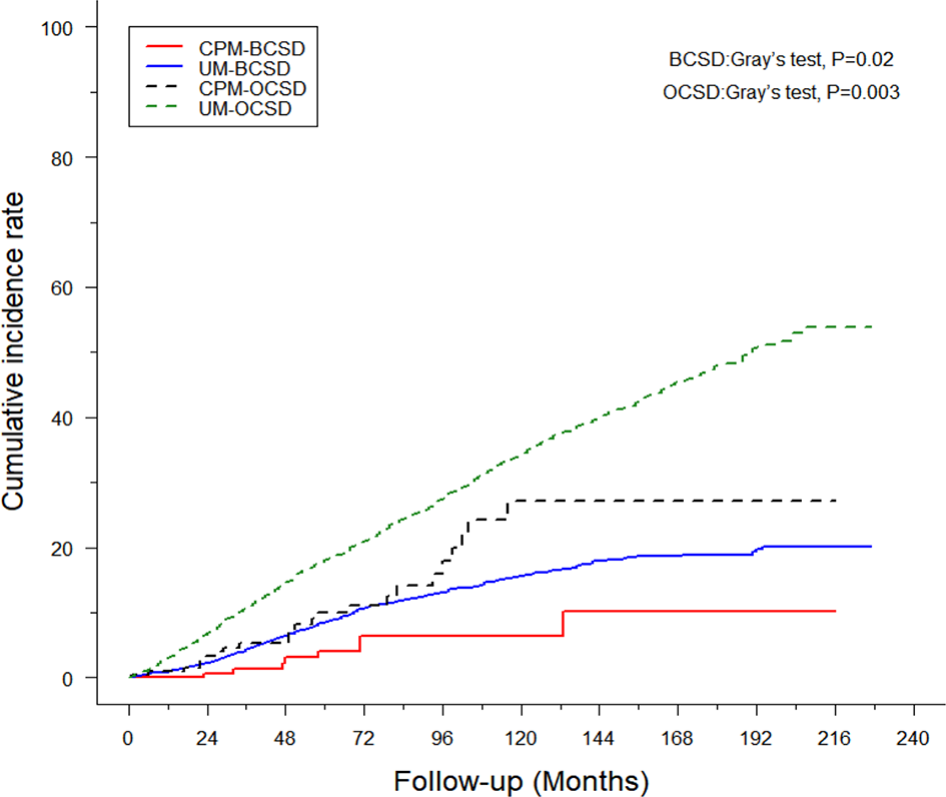
Cumulative incidence of breast cancer-specific death (BCSD) and other cause-specific death (OCSD) in the CPM group and UM group.

Of the 1,757 deaths from 4,405 patients, the whole cumulative incidence of BCSD was only 11.99% (528/4,405), but the cumulative OCSD (other cause-specific death) incidence was as high as 27.9% (1,229/4,405). In the univariate analysis by a competing risk model (**Table 3**), twelve variables (age, race, tumor grade, histology, T-stage, N-stage, radiation, chemotherapy, surgery, ER status, PR status, marital status), the *P*-value of which presented less than 0.05, were screened for competing risks regression analysis. Patients in the CPM group had both lower cumulative BCSD incidence (Gray’s test, P=0.02) and OCSD incidence (Gray’s test, P=0.003) than those in the UM group (**Figure 3**).

**Table 3:** Univariate competing risk model analysis of death causes in MaBC patients.

ITEMS	BCSD					OCSD				
	Event (n)	3-year (%)	5-year (%)	8-year (%)	*P-value*	Event	3-year (%)	5-year (%)	8-year (%)	*P-*value
**Age**					0.002					<0.001
≤65	271	3.8	7.9	13.8		247	4	6.8	11	
>65	257	4.6	8.8	12.1		982	15.7	26.5	40.1	
**Race**					0.002					0.02
White	423	4	7.7	12.1		1045	10.6	18.1	27.4	
Black	87	6.9	13.5	18.6		147	11.8	18.3	29.1	
Other/unknown	18	1.6	6.2	12.1		37	6.9	11.5	15.7	
**Grade**					<0.001					0.96
I	27	1.4	2.3	4.8		160	10.7	16.3	25.5	
II	231	2.9	6.4	10.4		635	10.4	17.4	27.4	
III or IV	270	7.2	13.5	19.5		434	10.7	18.9	27.3	
**Histology**					0.002					0.88
Infiltrating duct carcinoma	473	4.5	9	13.6			10.5	17.7	27.2	
Other	55	3	4.8	8.7		182	11.1	18.3	26.6	
**AJCC 6th T**					<0.001					<0.001
T0-1	147	1.5	3.3	6.8		595	8.8	15.8	25.4	
T2	284	5.9	12.5	18.2		486	11.1	18.5	27.5	
T3	26	12.2	17.3	24.5		30	12.1	16.8	25.4	
T4	71	12.1	18.4	23.6		118	19.7	29.3	38.1	
**AJCC 6th N**					<0.001					<0.001
N0	160	2.5	4.4	7.2		748	10.9	18.8	29	
N1	183	4.1	9.2	15.1		333	10.8	17.4	25.3	
N2	104	8.5	17.6	25.3		110	9.7	17	27.5	
N3	81	17.9	31.3	40.9		38	6.1	10.9	15.3	
**Surgery**					0.02					0.003
UM	520	4.4	8.5	13.1		1206	10.8	18.1	27.5	
CPM	8	1.3	4.1	6.4		23	5.4	10.1	17.9	
**Radiation**					<0.001					<0.001
Yes	197	6.1	13.3	19.9		243	6.9	13.3	21.9	
No	331	3.7	6.9	10.7		986	11.7	19.2	28.7	
**Chemotherapy**					<0.001					<0.001
Yes	293	5	10.9	17.7		282	4.5	8.5	14.5	
No	235	3.8	6.8	9.9		947	14.3	23.5	34.8	
**ER status**					0.04					0.08
Negative	22	14.3	16.7	19.3		33	16.3	19.8	29.2	
Positive	465	3.9	8	12.7		1063	10.1	17.3	26.6	
Unknown/other	41	4.8	9.9	12.9		133	14.4	23.1	32	
**PR status**					0.001					0.1
Negative	84	6.5	11.1	17.4		128	12.2	17.6	22.5	
Positive	393	4	7.8	12.4		944	10	17.4	27.2	
Unknown/other	51	4.7	9.7	12.4		157	13.6	21.5	31.6	
**Marital status**					<0.001					<0.001
Married	324	3.2	6.6	11		817	9.3	16.4	25.1	
Single	192	7.2	13.5	18.7		368	13.4	21.7	32.8	
Unknown	12	1.8	5.9	7.2		44	12.9	17.5	24.4	

SEER, Surveillance, Surveillance, Epidemiology, and End Results; AJCC, American Joint Committee on Cancer; ER, estrogen receptor; PR, progesterone receptor; BCSD, breast cancer-specific death; OCSD, other cause-specific death; UM, unilateral mastectomy; CPM, contralateral prophylactic mastectomy.

In the multivariate analysis by competing risks regression, the results suggested that histology and six variables (tumor grade, T-stage, N-stage, surgery, and marital status) were still the independent predictive factors of BCSD (**Table 4**). Results showed that CPM was significantly associated with better BCSD (HR=0.44, 95%CI: 0.22-0.88, P=0.02). In addition, patients with highly differentiated (grade I), T0-I stage, and N0 stage tumors, other histology, and those who were married tended to have significantly better BCSD than the corresponding group (*P*<0.05).

**Table 4:** Competing risks regression of BCSD.

Characteristics	BCSD		
	Hazard ratio	95% CI	*P*-value
**Age**	0.99	0.98-1.01	0.12
**Race**			
White	as reference		
Black	1.21	0.95-1.54	0.13
Other/unknown	0.81	0.49-1.3	0.38
**Histology**			
Other	as reference		
Infiltrating duct carcinoma	1.38	1.04-1.83	0.03
**Grade**			
I	as reference		
II	1.58	1.06-2.36	0.02
III or IV	2.27	1.52-3.4	<0.001
**AJCC 6th T**			
0-I	as reference		
II	1.95	1.6-2.4	<0.001
III	2.62	1.71-3.99	<0.001
IV	2.19	1.62-2.97	<0.001
**AJCC 6th N**			
0	as reference		
I	1.81	1.44-2.27	<0.001
II	2.85	2.16-3.76	<0.001
III	4.99	3.68-6.78	<0.001
**Surgery**			
UM	as reference		
CPM	0.44	0.22-0.88	0.02
**Radiation**			
No	as reference		
Yes	0.99	0.8-1.21	0.89
**Chemotherapy**			
No	as reference		
Yes	1.14	0.91-1.41	0.25
**ER status**			
Unknown/other	as reference		
Negative	1.19	0.5-2.81	0.69
Positive	0.87	0.44-1.73	0.70
**PR status**			
Unknown/other	as reference		
Negative	1.48	0.77-2.84	0.24
Positive	1.09	0.59-2.02	0.78
**Marital status**			
Married	as reference		
Single	1.35	1.12-1.63	0.002
Unknown	0.79	0.45-1.39	0.41

SEER, Surveillance, Surveillance, Epidemiology, and End Results; AJCC, American Joint Committee on Cancer; ER, estrogen receptor; PR, progesterone receptor; BCSD, breast cancer-specific death; UM, unilateral mastectomy; CPM, contralateral prophylactic mastectomy.

### Construction of Competing Risks Regression Nomogram Model

**Figure 4:**
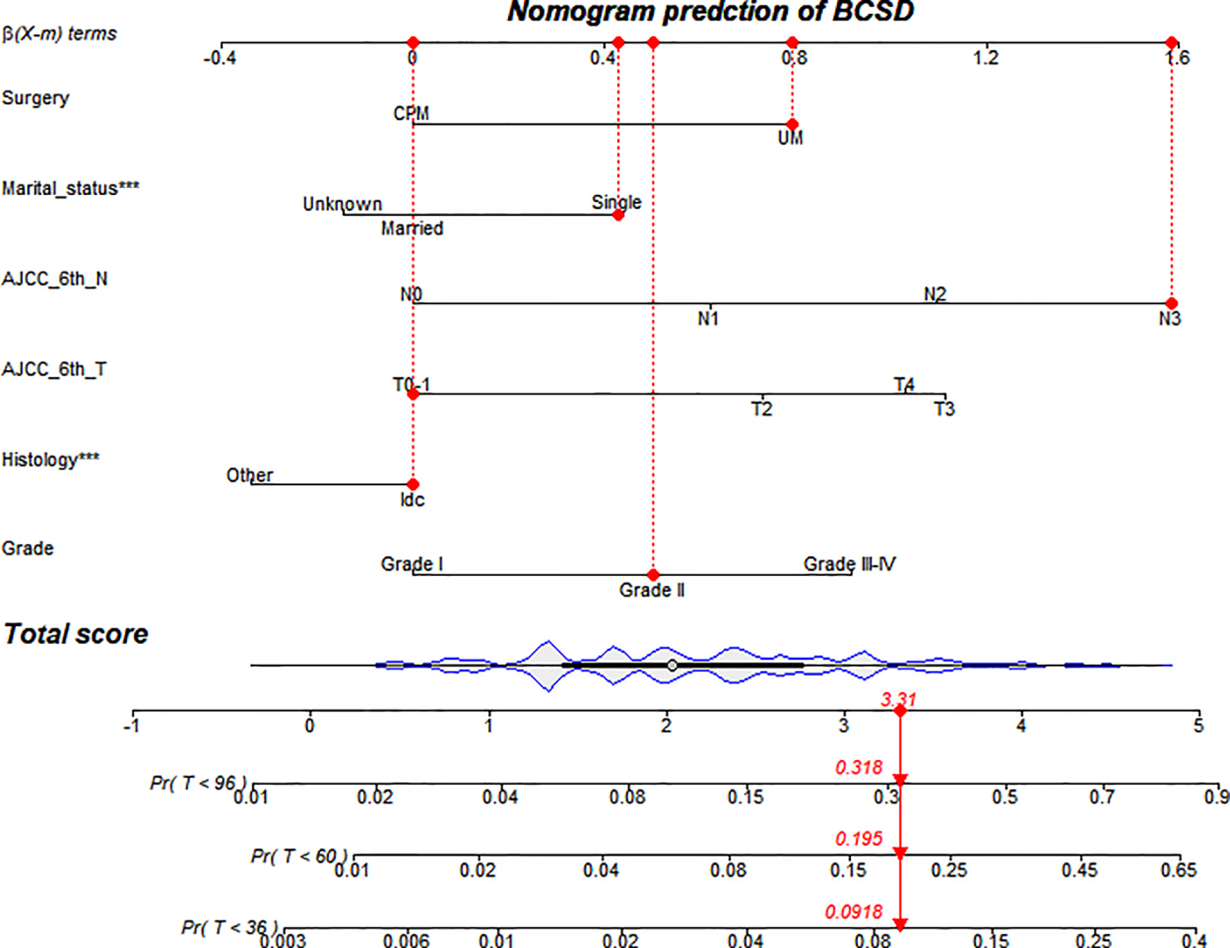
Competing risks regression nomogram model for MaBC patients.

Based on screening variables, the nomogram model established by competing risks regression models was used for forecasting the BCSD of every patient after three, five, and eight years, adjusted variables pointed to a score deriving from the scale, then we could get a total score by adding up all scores (**Figure 4**). The predictive cumulative probabilities of BCSD after three, five, and eight years could be evaluated by the total score according to the bottom scale. By using the nomogram, we forecasted a given patient after three, five, and eight years a BCSD of 9.2%, 19.5%, and 31.8%, respectively.

### Clinical Value of the Nomogram Compared With the AJCC-TNM Stage

**Figure 5:**
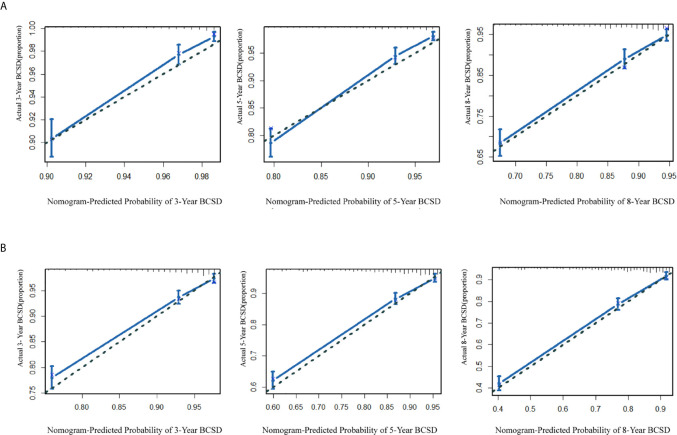
**(A)** The calibration curve for predicting patient BCSD after three, five, and eight years in the training cohort; **(B)** the calibration curve for predicting patient BCSD after three, five, and eight years in the internal validation cohort.

A portion of the cohort (30%) was chosen at random for internal validation. As shown in **Table 5**, the C-index was 0.76 (95%CI: 0.75-0.77) in the training cohort and 0.75 (95%CI: 0.74-0.77) in the validation cohort, implying improved prediction capability compared with AJCC-TNM stage (training cohort: 0.72, 95%CI, 0.71-0.73; validation cohort: 0.69, 95% CI, 0.69-0.72, respectively). Calibration curves also reflected the favorable consistency between nomogram-predicted and observed BCSD at 3-year, 5-year, and 8-year intervals (**Figures 5A**, **B**).

**Table 5:** C-index, NRI, and IDI of the nomogram and AJCC-TNM stage system in BCSD prediction for MaBC patients.

	Training cohort		Validation cohort	
NRI (*vs.* the AJCC criteria-based tumor staging)	Estimate	95% CI	Estimate	95% CI
For 3-year BCSD	0.54	0.31-0.69	0.51	0.07-0.83
For 5-year BCSD	0.55	0.27-0.67	0.45	0.02-0.74
For 8-year BCSD	0.49	0.24-0.61	0.33	0.16-0.34
IDI (*vs*. the AJCC criteria-based tumor staging)				
For 3-year BCSD	0.02	0.01-0.03	0.02	0.003-0.04
For 5-year BCSD	0.03	0.01-0.04	0.04	0.01-0.07
For 8-year BCSD	0.04	0.02-0.06	0.04	0.004-0.04
C-index				
The nomogram	0.76	0.75-0.77	0.75	0.74-0.77
AJCC-TNM stage system	0.72	0.71-0.73	0.71	0.69-0.72

AJCC, American Joint Committee on Cancer; BCSD, breast cancer-specific death.

The NRI and IDI were also performed to compare the efficiency between the nomogram and AJCC-TNM stage (**Table 5**). In the training cohort, the NRI values for the 3-year, 5-year, and 8-year BCSD were 0.54 (95%CI: 0.31-0.69), 0.55 (95%CI: 0.27-0.67), and 0.49 (95%CI: 0.24-0.61), respectively, the IDI values for the 3-year, 5-year, and 8-year BCSD were 0.02 (95%CI: 0.01-0.03), 0.03 (95%CI: 0.01-0.04), and 0.04 (95%CI: 0.02-0.06), respectively. While using the nomogram in the validation cohort, the NRI values for the 3-year, 5-year, and 8-year BCSD were 0.51 (95%CI: 0.07-0.83), 0.45 (95%CI: 0.02-0.74), and 0.33 (95%CI: 0.16-0.34), respectively, the IDI values for the 3-year, 5-year, and 8-year BCSD were 0.02 (95%CI: 0.003-0.04), 0.04 (95%CI: 0.01-0.07), and 0.04 (95%CI: 0.004-0.04), respectively. In summary, the abovementioned results suggested that the competing risks regression nomogram model had significantly enhanced precision and reliability for BCSD prediction compared with the TNM stage system.

## Discussion

In this retrospective study, we conducted Cox regression models and competing risk analysis based on 4,405 male patients with non-metastatic breast cancer in the SEER database from 1998 to 2015. The application was significantly associated with better BCSS and BCSD. Based on the corresponding parameters by competing risks regression, we built a nomogram to predict the 3-year, 5-year, and 8-year breast cancer-specific death (BCSD). To our knowledge, this was the first and largest population-based nomogram model to predict the impact of CPM on MaBC by competing risk analysis.In our study, surgery procedure was associated with improvement in BCSS and OS, which were objective and bias-free measurements for patients with MaBC. In the Kaplan-Meier curve analysis, significant improvements in BCSS and OS were observed in the CPM group rather than the UM group. To reduce the estimation bias and further investigate the efficiency of CPM on BCSS and OS for patients with MaBC, the multivariate Cox regression models analysis was performed. After adjusting for demographic, clinicopathological, and therapeutic variables, we found that administration of CPM could prolong BCSS, but had the threshold value of benefit in OS in comparison with UM. These findings were inconsistent with the previous trials where the application of CPM played a vital role in MaBC treatment (18–20). Multiple single and multi-institution studies reported the CPM’s positive effect on OS and disease-free survival (DFS). Four single (21, 22) and three multi-institution (23–25) studies demonstrated that CPM could have benefit in DFS, while two single (21, 26) and three multi-institution (23–25) studies indicated an OS benefit. A recent review study showed that patients who received CPM might be more healthy and had access to more advanced treatments than patients who did not undergo CPM (27).

To eliminate the estimation bias from other causes of death and further investigate the efficacy of CPM on BCSD, competing multivariable regression models analysis which is common in oncology research was performed (28–31). After performing competing risks regression, we found that the patients in the CPM group had better BCSD in comparison with the UM group. The main reasons might be that most of the research involving CPM was conducted in patients with FBC rather than patients with MaBC. Several studies concentrated on the prevention of contralateral breast cancers (CBCs) through the administration of CPM (20, 23, 32, 33). And BRCA mutation carriers, who had a high risk of CBCs, also obtained a survival benefit from CPM (34–36). Many patients consequently tended to select CPM to reduce the risk of CBCs. Many studies have shown that CBCs tend to have more favorable tumor features, and patients who develop CBCs in a short interval from their primary cancer have worse prognosis than those who develop CBCs at a longer interval, especially in young patients with large tumors, and those who are node-positive (37–41). However, it is controversial whether worse survival is caused by the CBCs, which represents the aggressive biology of the primary tumor, distant metastatic disease, and older, inferior systemic treatments.

In addition, MaBC and FBC have different biological characteristics, such as the rate of ER-positive tumors and age at diagnosis. In our study, the rate of ER and PR-positive tumors were as high as 90.8% and 81.2%, respectively, but the percentage of ER-positive tumors and PR-positive tumors in FBC patients were only 78% and 64% in a previous study (42, 43). The majority of male cases who developed BC were older than those in FBC. Previous research reported that MaBC tended to have a 1.75 times higher risk of distant metastasis than FBC (7% *vs.* 4%) (44, 45). Furthermore, patients with MaBC were likely to have a higher mutation rate of CHEK2 c.1100delC and BRCA2, which play a particularly prominent role in metastasis and the prognosis of disease, than those in FBC (11, 12, 46–49). In brief, MaBC patients were more likely to have poorer differentiated grade, were older, a higher node-positive, higher rates of lymphovascular invasion, and estrogen receptor (ER+) tumors. Therefore, there are differences in treatment procedures, for example chemotherapy/radiotherapy and the corresponding prognosis between MaBC and FBC.

Meanwhile, this study set up a nomogram model to predict BCSD in patients with MaBC. After integrating the demographic and clinicopathological characteristics, the nomogram model could be more precise than the conventional TNM stage system, such as the AJCC stage system. In the traditional sense, the AJCC-TNM stage system was the preferred alternative for predicting the prognosis of patients with carcinoma. In general, the stages of this system were strongly correlated with BCSD (50). Inevitably, patients at the same stage often had different prognoses. The underlying reasons might be the vagueness in the TNM-stage system and the variables which were not included in the sociodemographic characteristics, such as age, marital status, and so on. Actually, in our study, married patients with a well-differentiation level and T0-1 stage and N0 stage tumors tended to have better prognostic indicators for BCSD. These results are consistent with previous reports (18–20, 35, 46, 47) and indicate that both demographic and clinicopathological characteristics, such as marital status and tumor differentiation level, were objective and reliable prognostic indicators in men with breast carcinoma. Then, the NRI value and IDI value of the nomogram confirmed that the nomogram had better prediction power than the AJCC-TNM stage system. Furthermore, the favorable results were replicated well in the validation cohort. In summary, the nomogram could provide precise and personalized prediction of the cumulative risk in patients with MaBC after CPM.

Our subject indeed has limitations, as shown below: Firstly, studies that randomly assigned patients into different groups by treatment methods were needed. The retrospective study could not prove causation and may be subject to selection bias and uncontrolled confounding factors, even with the administration of competing risks regression models. Secondly, we were unable to avoid the possibility that the observed risks reduction might exclude the influence of potential confounders, such as family history, insurance coverage, comorbidities, health status, MRI application, patient anxiety, BRCA gene status, counseling, and so on. These data greatly impacted the clinical decisions and even breast cancer prognosis (18–20, 34–36, 46, 47). Thirdly, there was a big gap between CPM and UM that may have some bias to the data, and the study sample might be insufficient to uncover some differences in the abovementioned phenomenon. Next, the proportion of T1 stage (49.5%), T2 stage (41.1%), N0 stage (56.2%), and N1 stage (29.6%) may have been too high in our study, this statistical bias from the SEER database might lead to the result that the efficacy of radiotherapy and chemotherapy were limited in our study. Randomized controlled clinical and multicenter-clinical trials with long follow-up periods are still needed to further confirm this. Lastly, *P* value <0.05 was used to possess the statistics sense, and no adjustment was made for multiple analysis; the chance of falsely rejecting a null hypothesis may exceed 0.05.

## Conclusion

The administration of CPM was associated with the decrease in risk of BCSD in patients with MaBC. The nomogram could provide precise and personalized prediction of the cumulative risk in patients with MaBC after CPM. Randomized controlled clinical and multicenter-clinical trials with long follow-up time are still needed to further confirm the effects of CPM on BCSD and the prediction efficacy of the nomogram.

## Data Availability Statement

The datasets presented in this study can be found in online repositories. The names of the repository/repositories and accession number(s) can be found below: https://seer.cancer.gov.

## Author Contributions

KL and BW drafted the manuscript and analyzed data, ZY, HC, and YL generated the figure, and RY performed the background research. CZ and JH edited the manuscript. All authors have read and approved the content of the manuscript.

## Funding

This study was supported by the National Natural Science Foundation of China (NSFC 81502413 to CZ and NSFC 81702633 to BW) and the Shan’xi Provincial Natural Science Foundation of China (SNSFC 2019SF-145 to CZ).

## Conflict of Interest

The authors declare that the research was conducted in the absence of any commercial or financial relationships that could be construed as a potential conflict of interest.
